# Iron chelators reverse organ damage in type 4B hereditary hemochromatosis: Case reports

**DOI:** 10.1097/MD.0000000000025258

**Published:** 2021-04-02

**Authors:** Ling-yan Wu, Zhen-ya Song, Qing-hai Li, Li-jun Mou, Ying-ying Yu, Si-si Shen, Xiao-xiao Song

**Affiliations:** a Department of General Practice; b Department of Radiology; c Department of Nephrology; d Department of Endocrinology, Second affiliated Hospital of Zhejiang University, HangZhou, Zhejiang Province, China.

**Keywords:** gain of function, hereditary hemochromatosis, iron chelator, secondary diabetes, solute carrier family 40 member 1 mutation

## Abstract

**Rationale:**

Hereditary hemochromatosis (HH) is a hereditary disorder of iron metabolism. It is classified into 4 main types depending on the underlying genetic mutation: human hemochromatosis protein (HFE) (type 1), hemojuvelin (HJV) (type 2A), HAMP (type 2B), transferrin receptor-2 (TFER2) (type 3), and ferroportin (type 4). Type 4 HH is divided into 2 subtypes according to different mutations: type 4A (classical ferroportin disease) and type 4B (non-classical ferroportin disease). Type 4B HH is a rare autosomal dominant disease that results from mutations in the Solute Carrier Family 40 member 1 (SLC40A1) gene, which encodes the iron transport protein ferroportin.

**Patient concerns:**

Here we report 2 elderly Chinese Han men, who were brothers, presented with liver cirrhosis, diabetes mellitus, skin hyperpigmentation, hyperferritinaemia as well as high transferrin saturation.

**Diagnosis:**

Subsequent genetic analyses identified a heterozygous mutation (p. Cys326Tyr) in the SLC40A1 gene in both patients.

**Interventions:**

We treated the patient with iron chelator and followed up for 3 years.

**Outcomes:**

Iron chelator helped to reduce the serum ferritin and improve the condition of target organs, including skin, pancreas, liver as well as pituitary.

**Lessons:**

Type 4B HH is rare but usually tends to cause multiple organ dysfunction and even death. For those patients who have difficulty tolerating phlebotomy, iron chelator might be a good alternative.

## 1 Introduction

Hereditary hemochromatosis (HH) is a genetic disorder characterized by iron deposition and tissue injury in multiple organs. According to different gene mutations, HH was divided into 4 types.^[1–5]^ Type 4B HH, which was considered as rare but severe, usually developed a phenotype with hyperferritinemia, high transferrin saturation iron, and iron deposition in hepatic parenchyma and other important tissues, resulting in skin hyperpigmentation, liver cirrhosis, secondary diabetes, central hypothyroidism, central hypoandrogenism, and so on.^[1]^ C326 mutation is considered to be one of the most severe forms of ferroportin mutation, with 3 types of mutations published till now^[6–8]^ (Table 1). All these papers were published for novel mutations, while the treatment and prognosis were rarely mentioned. Here, we report 2 brothers in a Chinese Han population, with a heterozygous mutation (p.Cys326Tyr) in the solute carrier family 40 member 1 (SLC40A1) gene, both suffered from severe iron overload complications, including liver cirrhosis, diabetes mellitus, and skin hyperpigmentation. After a 3-years follow-up, we found that deferasirox is effective in reversing organ damage in patients cannot tolerate phlebotomy, and improve patient's life quality.

**Table 1:** Currently recognized C326 mutations.

Site	Author	Year	Iron dep	Ts	Sf	Treatment
Cys326Tyr^[6]^	Viprakasit V	2004	Not mentioned	Elevated	Not mentioned	Not mentioned
Cys326Ser^[7]^	Sham RL	2005	Hepatocyte	Elevated	Elevated	Roughly mentioned
Cys326Phe^[8]^	S-R Chen	2015	Hepatocyte skin pancreas et	Elevated	Elevated	Not mentioned

Sf = serum ferritin, Ts = transferrin saturation.

### 1.1 Clinical report

#### 1.1.1 Patient A

**Figure 1:**
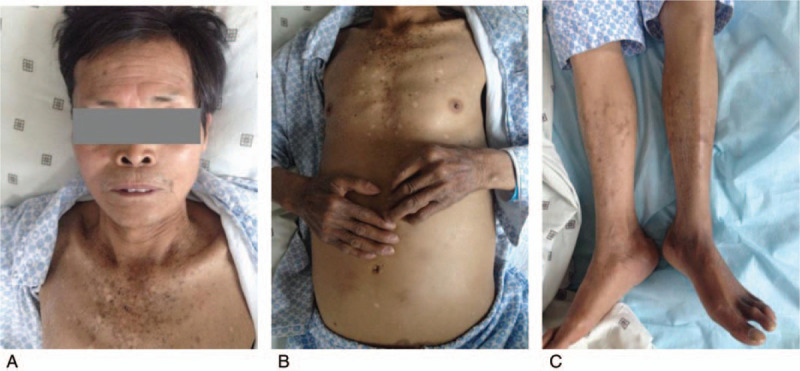
Systemic skin hyperpigmentation, especially the chest, hands, and feet.

**Figure 2:**
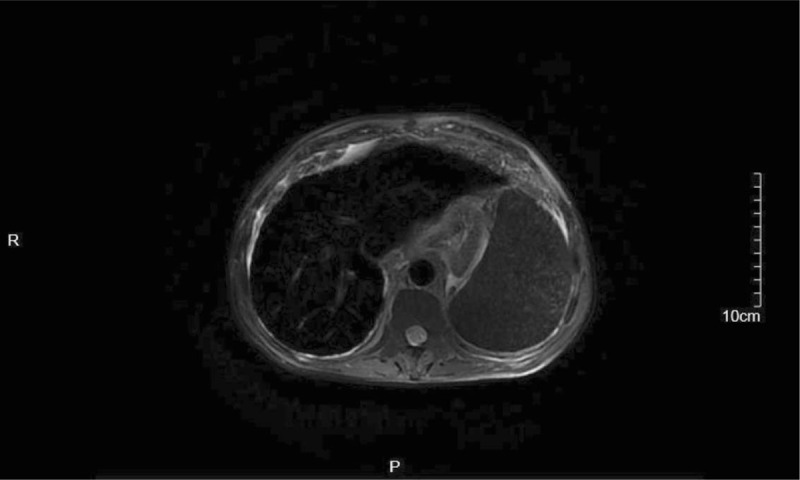
Transverse T2 Weighted image (T2WI) with respiratory gating and fat saturation showed that the signal intensity of liver and spleen was significantly reduced due to iron deposition.

**Figure 3:**
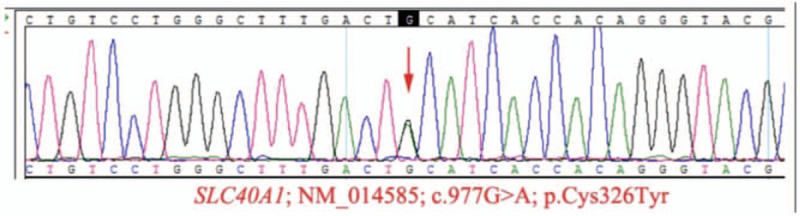
Detection of heterozygous mutation in EX7/CDS7 of SLC40A1. SLC40A1 = solute carrier family 40 member 1.

A 65-year-old man presented to our hospital complaining of weight loss and fatigue for the previous 2 years. Five years previously, he had been diagnosed with liver cirrhosis, and he had a 2-year history of diabetes, which was well-controlled by insulin. He had been admitted to another hospital for fatigue and dropsy 2 months prior to presentation at our hospital, where he was diagnosed with decompensated liver cirrhosis and heart failure; his condition stabilized after treating with insulin, albumin, and diuretics. He had smoked for 40 years with 40 cigarettes per day, had quit 1 year previously, and did not drink alcohol. He had no history of blood transfusion, hemolysis, or oral iron supplementation. Physical examination revealed skin hyperpigmentation (Fig. 1) and hepatosplenomegaly. Blood testing showed a very high serum ferritin concentration (>1650 μg/L). The patient had liver dysfunction, secondary diabetes, central hypothyroidism, central hypoandrogenism, and low growth hormone (Table 2). HIV antibody, hepatitis viral-related serum antibody (hepatitis A, B, C, D, and E virus, and Epstein–Barr virus), and autoimmune hepatitis antibody testing were negative. The Magnetic Resonance Imaging (MRI) scan showed iron deposition in the liver and spleen (Fig. 2), liver cirrhosis, splenomegaly, and ascites. Fibroscan indicated liver stiffness of 53.9 (KPA). A gastroscopy showed esophageal and gastric varices and portal hypertensive gastropathy. Doppler echocardiography revealed heart failure with reduced ejection fractions (EF) (48.3%). Liver biopsy was a high-risk procedure for the patient, as his thrombocytopenia and bleeding tendency. Blood samples from the patient, as well as from his 2 sisters and the youngest brother, his 2 daughters, the daughter of his elder daughter, were then sent for genetic testing for HH. Written informed consent was obtained from the patient and his relatives. We detected a heterozygous mutation in EX7/CDS7 of SLC40A1 (Fig. 3). Regarding the patient's family history, his father had died due to myocardial infarction and his mother had died from diabetes. He had 3 younger brothers and 2 younger sisters, all alive. He had 2 daughters. The same mutation in SLC40A1 was detected in his youngest brother, his 2 younger sisters, and both of his daughters, as well as in the daughter of his elder daughter. His other 2 younger brothers refused to undergo genetic testing. The patient's elder daughter, who was 39 years old had skin hyperpigmentation, hyperferritinemia (>1650 μg/L), and high transferrin saturation (59.6%). Due to the patient's anemia, thrombocytopenia, liver dysfunction, and hyperglycemia, we did not treat him with phlebotomy therapy. We suggested deferasirox and liver transplantation to him but he did not agree with this suggestion. However, the patient died of massive hemorrhage of the upper gastrointestinal tract 2 months after discharge.

**Table 2:** Target organs damaged by iron deposition.

	Gender	Age	Skin	Liver	Spleen	Pancreas	Heart	Pituitary	Gonad	Adrenal	Thyroid	Marrow
Patient A	Male	70	+	+	+	+	+	+	+	N	+	N
Patients B	Male	57	+	+	+	+	−	+	+	−	−	−

+ = target organs damaged by iron deposition, − = target organs not affected by iron deposition, N = unidentified.

#### 1.1.2 Patient B

**Figure 4:**
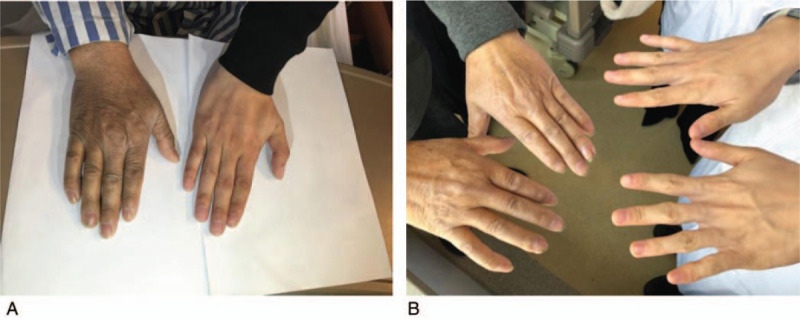
(A) Patient (left) has severe skin hyperpigmentation before treatment. (B) The skin color of the patient (left) was almost the same with others of his age after treatment.

**Figure 5:**
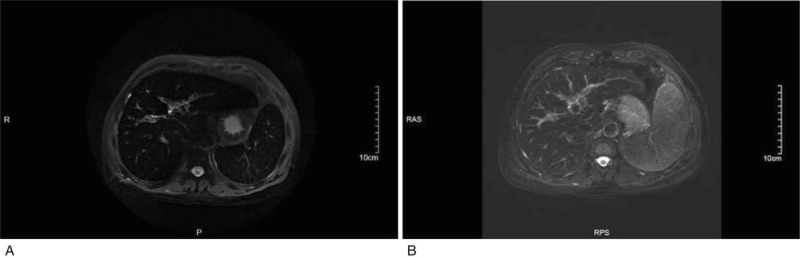
Transverse T2WI using respiratory gating and fat suppression techniques showed that the signal intensity of the liver and spleen recovered after treatment (B) compared with the pretreatment (A), suggesting a significant reduction of iron deposition in the liver and spleen.

A 54-year-old man, the youngest brother of the former patient, complained of dry mouth, polydipsia, and polyuria for the previous 1 month, as well as skin hyperpigmentation for >10 years. He was admitted to the local hospital for hepatocirrhosis ascites a week prior to admission in our institution. He had 1 daughter and 1 son. The same mutation in SLC40A1 was detected in his daughter, but his son refused to undergo genetic testing. The patient did not smoke or drink alcohol. Physical examination revealed skin hyperpigmentation (Fig. 4) and mild hepatomegaly. Blood testing showed extremely high serum ferritin concentration (10175.2 μg/L) and high transferrin saturation (74.9%). His liver function was basically normal, but he had low white blood cell count, thrombocytopenia, and anemia. His blood sugar was high, and his C-peptide level was low (0.17–0.16–0.17–0.25–0.34 nmol/L). Additionally, the patient had central hypoandrogenism. His serum Insulin-like Growth Factor-1 (IGF-1) (37.3 ng/mL) and Insulin-like Growth Factor binding protein-3 (IGFBP-3) (1.24 μg/mL) were low. However, his thyroid hormones, cortical hormones, and adrenocorticotropic hormone (ACTH) were in the normal range. HIV antibody, hepatitis viral-related serum antibody (hepatitis A, B, C, D, and E virus, and Epstein–Barr virus), and autoimmune hepatitis antibody testing were negative. An MRI scan showed iron deposition in the liver and spleen (Fig. 5). The patient refused to undergo liver biopsy. He agreed to start phlebotomy therapy (200 mL per week) and deferasirox (20 mg/kg/d). And he was also treated with insulin for his secondary diabetes. His serum ferritin decreased steadily. After his sixth phlebotomy treatment, his blood platelet level dropped to 16 × 10^9^/L, and he refused subsequent phlebotomy treatments. However, he continued taking deferasirox (20 mg/kg/d) for 26 months. His serum ferritin decreased to 480.9 μg/L. After that, the patient quit deferasirox for personal reasons but continued his follow-up visits. We admitted the patient for reexamination 6 months after he stopped taking deferasirox. The patient reported feeling healthy during the previous 2 years. His skin hyperpigmentation had faded, and his appearance was the same as that of healthy people his age (Fig. 4). A blood test showed his serum ferritin was 555.9 μg/L, and his transferrin saturation was 94.6%. His blood platelet level was 54 × 10^9^/L, and his white blood cell count and hemoglobin were slightly below the normal range. His blood sugar was well-controlled under about half of the former dosage of insulin as his first admission, and his C-peptides seemed to be slightly improved (0.18–0.20–0.28–0.51–0.57 nmol/L). His serum IGF-1 and IGFBP-3 were also elevated. However, his serum testosterone was lower than before. His thyroid hormones, cortical hormones, and ACTH were still in the normal range. His bone marrow biopsy indicated active hematopoiesis, meaning that his thrombocytopenia was a result of hypersplenism due to cirrhosis. An MRI scan of the liver and spleen also indicated obvious improvement compared with former scans (Fig. 5) (Table 3).

**Table 3:** Improvement of target organs after treatment.

	Sf, μg /L	Ts (%)	Skin	Liver (MRI)	Spleen (MRI)	Pancreas (C-peptide)	Pituitary (IGF-1/IGFBP-3)	Gonad (testosterone)
Before	10,175.2	74.9	Dark					
After	555.9	94.6	Almost normal	Improved	Improved	Elevated	Elevated	Reduced

IGF-1 = insulin-like growth factor-1, IGFBP-3 = insulin-like growth factor binding protein-3, MRI = magnetic resonance imaging, Sf = serum ferritin, Ts = transferrin saturation.

## 2 Discussion

HH is defined as systemic iron overload caused by genetic mutations, which generally lead to a deficiency of hepcidin, including decreased production of hepcidin or decreased activity of hepcidin-ferroportin binding.^[1–6]^ Homozygous mutations (specifically, the C282Y mutation) are the most common mutations in the HFE gene, which plays a role in hepcidin regulation. This mutation is found almost exclusively in white populations and leads to human hemochromatosis protein (HFE)-associated HH (also known as type 1 HH). Other genetic mutations, including HAMP (encoding hepcidin), HJV (encoding hemojuvelin), TFR2 (encoding transferrin receptor protein 2), and SLC40A1 (encoding ferroportin), can also cause HH; these are referred to as non-HFE HH.^[2,4,9]^ Both HFE-associated HH and non-HFE-associated HH lead to hepcidin deficiency, resulting in uncontrolled ferroportin activity, increased iron release from enterocytes and macrophages into the plasma, and increased iron transport into parenchymal cells (particularly hepatocytes, pancreatic cells, and cardiomyocytes).^[1,10,11]^

Ferroportin is the only identified cellular iron exporter, which is highly expressed on the basolateral membrane of enterocytes and the plasma membrane of macrophages.^[12]^ Hepcidin is an iron-regulated peptide secreted by hepatocytes that regulates the activity of ferroportin.^[13]^ Hepcidin binds to ferroportin and induces its internalization and degradation, resulting in decreased iron efflux from cells into plasma. The negative feedback regulation of hepcidin to ferroportin activity maintains iron homeostasis.

Autosomal-dominant mutations of the ferroportin gene lead to impaired iron-exporting function or deficiency in hepcidin-ferroportin interactions, eventually resulting in iron overload^[14]^; this type is known as type 4 HH, and is also called ferroportin disease.^[15–17]^ Type 4 HH is divided into 2 subtypes according to different mutations: type 4A (classical ferroportin disease) and type 4B (non-classical ferroportin disease).^[1,4,5,15,18,19]^ Mutations that lead to the inability of ferroportin to present normally at the cell surface or create defective iron export activity (loss of function) are defined as type 4A HH^[4,5,15,19,20]^ and are characterized by hyperferritinemia, normal transferrin saturation, and iron overload in macrophages. Type 4B HH is caused by mutations that lead to the prevention of hepcidin-mediated internalization and the degradation of ferroportin (gain of function). This type is characterized by additional hepatocellular iron deposits and high transferrin saturation.^[4,5,15,19–22]^ However, as the phenotype and treatment of classical ferroportin disease differ from other types, some researchers do not consider these to be HH.^[1]^

C326 mutation, considered to be one of the most severe forms of ferroportin mutation, usually develops a phenotype with increased duodenal iron absorption, hyperferritinemia, high transferrin saturation, and iron deposition in hepatic parenchyma and other tissues.^[6–8,23]^ The ferroportin C326 residue is located in the hepcidin binding domain (HBD) of ferroportin, which is near the predicted cytosolic loop containing the 2 adjacent tyrosines that are phosphorylated in response to hepcidin binding.^[21,24–26]^ Mutations of C326 residue render ferroportin resistant to the inhibitory effect of hepcidin by impairing the process of hepcidin-ferroportin binding.^[8]^ Thus, the ferroportin activity is unregulated resulting in high serum ferritin and iron deposition within parenchymal cells (mostly hepatocytes, but also pancreatic, pituitary, and cardiac cells), causing damage and disease to target organs.^[11,18]^

Although several mutations associated with type 4B HH have been reported, most of these are rare.^[1]^ Since 2015, a few cases of SCL40A1 mutations have been reported in Chinese populations^[15]^; however, the C326Y mutation, which was first reported in 2004,^[6]^ has not been identified in a Chinese patient. The present case report describes 2 patients with the C326Y mutation, as confirmed by genetic testing.

In this report, patient A had multiple organs that had varying levels of dysfunction, including decompensate cirrhosis, heart failure, secondary diabetes (from iron deposits in the pancreas), and secondary hypothyroidism and hypogonadism (from iron deposits in the pituitary gland). Unfortunately, the patient was too sick to be treated with phlebotomy or iron chelator, and he died from massive upper gastrointestinal bleeding.

In contrast, the clinical condition of patient B was much better. Although he was also diagnosed with liver cirrhosis, secondary diabetes, and hypogonadism at admission, he was able to initiate appropriate treatment. Regarding treatment, all international guidelines agree that excess iron should be treated with venesection/phlebotomy.^[4,5,15,27,28]^ Phlebotomy therapy achieves the intended results in 2 ways: first, blood loss directly reduces the hemoglobin store of iron, and second, it induces erythropoiesis, which mobilizes stored iron. In the long term, the optimum concentrations of serum ferritin in the body's iron stores are in the low to normal range (serum ferritin concentration of 50–100 μg/L).^[15,29]^ Erythrocytapheresis, although not recommended in current guidelines, is reported to be an effective treatment for HH.^[15,29–31]^ Additionally, iron chelators, such as deferasirox, can effectively eliminate iron excess in patients with HH,^[2,15,27,32,33]^ but the experience of using iron chelator in patients with HH is limited.

We treated patient B with phlebotomy therapy and deferasirox, and his condition was well-controlled. His serum ferritin concentration and transferrin saturation levels steadily decreased. However, his platelet count also decreased following phlebotomy therapy. After the sixth phlebotomy session, the patient's platelet count was down to 16 × 10^9^/L, and we discontinued phlebotomy therapy considering the risk of bleeding and according to the patient's decision. We continued with deferasirox for 26 months before the patient stopped taking the medicine for financial reasons. During the 26 months, the patient's condition was good, without severe liver dysfunction or gastrointestinal reactions. A comprehensive examination of his condition at 6 months after stopping the deferasirox revealed marked overall improvement, including skin hyperpigmentation, serum iron metabolism indices, insulin dosage, and serum C-peptide levels. Due to patient refusal of liver biopsy, we used MRI to assess iron concentrations in the target organs.^[34,35]^ The MRI scans of the liver and spleen showed obviously reduced iron deposition, and the results were consistent with the patient's clinical features.

The 2 cases described in this report, with the same disease but different outcomes, suggest several conclusions. First, mutations of C326 residue impairs the process of hepcidin-ferroportin binding, blocks the negative feedback regulation of hepcidin to ferroportin activity, which results in iron deposition within parenchymal cells, and then leads to multiple organ dysfunction and even death. Second, C326 mutation is considered as one of the most severe forms, and early detection and diagnosis are extremely important. Third, MRI is an excellent alternative to liver biopsy, as it is noninvasive and effective. Fourth, although phlebotomy therapy is still the main treatment for HH, this therapy may lead to thrombocytopenia. Finally, deferasirox are effective in treating type 4B HH, leading to at least partial recovery of the impaired target organs and reduced iron deposition. In conclusion, this rare disease in Asian population warrants clinical attention and future research.

## Author contributions

**Conceptualization:** Lingyan Wu, Xiaoxiao Song.

**Data curation:** Lingyan Wu, Sisi Shen.

**Funding acquisition:** Xiaoxiao Song.

**Methodology:** Qinghai Li, Li-jun Mou.

**Project administration:** Xiaoxiao Song.

**Resources:** Lingyan Wu, Yingying Yu.

**Supervision:** Zhenya Song.

**Writing – original draft:** Lingyan Wu.

**Writing – review & editing:** Lingyan Wu, Xiaoxiao Song.
